# Cost-optimized placenta-targeted nanoparticle for localized immune cloaking in recurrent pregnancy loss

**DOI:** 10.1530/RAF-25-0077

**Published:** 2025-10-14

**Authors:** Mohsen Dashti, Arvin Amir, Mehdi Yousefi

**Affiliations:** ^1^ Immunology Research Center, Tabriz University of Medical Sciences, Tabriz, Iran; ^2^ Department of Immunology, School of Medicine, Tabriz University of Medical Sciences, Tabriz, Iran; ^3^ iFertility Technologies, Melbourne, Australia; ^4^ Research Center for Integrative Medicine in Aging, Aging Research Institute, Tabriz University of Medical Sciences, Tabriz, Iran

**Keywords:** recurrent pregnancy loss, RPL, nanoparticle, placenta-targeted, immune cloaking

## Abstract

**Abstract:**

Recurrent pregnancy loss (RPL) is defined as the occurrence of two or more consecutive miscarriages and affects approximately 1–2% of reproductive-aged couples. Immune-mediated factors at the maternal–fetal interface are increasingly recognized as significant contributors to otherwise unexplained RPL. Current therapeutic approaches largely rely on systemic immunosuppression, which demonstrates limited efficacy and imposes substantial maternal risks. Here, we propose a drug-free, placenta-targeted nanoparticle (NP) system for localized immune cloaking utilizing well-characterized, cost-effective materials. The core design consists of a biodegradable PLGA matrix, a lipid–polyethylene glycol (PEG) stealth layer, superparamagnetic iron oxide nanoparticles (SPIONs) for imaging, and placental-homing peptides for targeted delivery. The mechanisms of immune cloaking may include PEG stealth, red blood cell membrane coating, or immunomodulatory ligands to induce site-specific immune tolerance while avoiding the adverse effects associated with systemic immunosuppression. We discuss material accessibility, feasibility of large-scale manufacture, and the preclinical evidence base to be developed. Finally, we outline regulatory pathways and prospective clinical trial designs. This localized NP-based treatment may offer a significant reduction in RPL incidence by promoting targeted maternal–fetal immune tolerance while addressing the safety and cost limitations inherent to current broad-spectrum immunotherapies.

**Lay summary:**

Miscarriage is heartbreaking and a growing issue that many families deal with. For some women, it occurs repeatedly for no apparent reason. One of the major causes is thought to be an overreactive immune response, in which the immune system of the mother unintentionally targets the growing fetus. Currently, medications that suppress the entire immune system, also known as immunosuppressive treatments, are occasionally administered to women who have experienced repeated miscarriage. These therapies may have systemic effects on the whole body, can be costly, and put the mother at higher risk of developing serious adverse events. Our study proposes a new, secure option. We recommend using nanoparticles, tiny particles specifically engineered to reach the placenta, and give details regarding the design, safety and efficacy protocols, and the road map to make this product commercially available. Once in place, the nanoparticles can help establish a secure environment where the fetus can grow safely by shielding the fetus from the mothers’ immune system. Nanoparticles are a growing treatment option in many fields and can also be used in reproductive medicine to help families who have suffered recurrent miscarriages. In addition, this could decrease the burden of miscarriage on both families and the health care system by improving pregnancy outcomes and reducing the need for dangerous medications.

## Introduction

Recurrent pregnancy loss (RPL) is a distressing reproductive condition defined as two or more consecutive miscarriages, affecting approximately 1–2% of couples (The ESHRE Guideline Group on RPL *et al.* 2023). Despite significant diagnostic evaluations, up to 50% of RPL cases remain unexplained (The ESHRE Guideline Group on RPL *et al.* 2023). An increasing body of evidence points to immune maladaptation at the maternal–fetal interface as a key factor in such unexplained cases (Zhang *et al.* 2018*a*, Xu *et al.* 2021). In the normal decidual immune environment, the semi-allogeneic embryo is tolerated; regulatory T cells (T-regs), which show suppressive functions mainly through cytokine secretion (e.g. IL-10, TGF-β) and cell–cell contact inhibition, together with tolerogenic macrophages that promote tissue remodeling and secrete anti-inflammatory mediators, collaborate with trophoblast cells to foster an immune-privileged niche (Zhang *et al.* 2018*a*, Bai *et al.* 2021, Yousefzadeh *et al.* 2022). Pregnancy loss can be associated with excessive maternal immune activation, and when these regulatory mechanisms fail, the embryo may be targeted (King *et al.* 2016, Xu *et al.* 2021).

In the current clinical setting, immunological RPL is treated with systemic immunosuppressant medications (e.g. corticosteroids), intravenous immunoglobulins (IVIGs), TNF inhibitors, and intradermal lymphocyte therapies (Parhizkar *et al.* 2022, Yang *et al.* 2022, Aslanian-Kalkhoran *et al.* 2023, The ESHRE Guideline Group on RPL *et al.* 2023, Dashti *et al.* 2025). The effectiveness of these interventions has been inconsistently demonstrated in clinical trials, and they can be quite expensive and associated with significant side effects (The ESHRE Guideline Group on RPL *et al.* 2023). While not necessarily enhancing live birth rates, such medications indeed disturb global immune function, raising the risk of infections (The ESHRE Guideline Group on RPL *et al.* 2023). Given the lack of solid evidence and notable adverse–event profiles, several professional bodies – including ESHRE – have questioned the value of broad immunotherapy for unexplained RPL (uRPL) (Practice Committee of the American Society for Reproductive Medicine 2012, The ESHRE Guideline Group on RPL *et al.* 2023).

Instead of suppressing the whole immune system, therapies for immunological RPL should target the maternal–fetal interface (Bai *et al.* 2021, Xu *et al.* 2021). Nanoparticles (NPs) show promise, as they have been used to systemically deliver chemotherapeutics and mRNA vaccines (Fam *et al.* 2020, Li *et al.* 2022). By using tissue-specific targeting ligands and surface modifications, NPs can be directed to particular organs, including the placenta, thereby reducing off-target effects (Li *et al.* 2022). Moreover, NPs can be manufactured without strong immunosuppressive medications, depending instead on surface engineering to produce a confined ‘immuno-privileged’ zone. This is conceptually similar to the natural mechanisms by which placental exosomes modulate maternal immune cells (Chen *et al.* 2022). A placenta-specific NP that cloaks the local immune environment may circumvent the safety concerns plaguing systemic therapies while reducing manufacturing costs and simplifying administration.

## Hypothesis

### Preclinical precedents for NP-mediated immune modulation in pregnancy

Exosome-based nanotherapeutics that target CD16^+^ decidual immune cells have been shown to significantly reduce miscarriage rates in a recent mouse model, demonstrating the potential of immune-cell-specific delivery systems (Wang *et al.* 2024). In non-human primates, uterine artery-mediated NP delivery of placenta-specific IGF-1 preserved fetal growth, setting a strong translational and mechanistic precedent (Schmidt *et al.* 2025). Additional rodent studies using MitoQ-loaded NPs demonstrated selective placental distribution and suppression of hypoxia-related fetal growth restriction without crossing into fetal tissues (Jiang *et al.* 2022). Finally, *ex vivo* human placenta models verified that cytotrophoblasts can efficiently internalize liposomal siRNA while protecting the fetus (Jiang *et al.* 2022).

Despite the lack of direct miscarriage prevention models using immune-modulatory NPs, relevant preclinical studies provide mechanistic feasibility. For example, lipid nanoparticles induced placental secretion of anti-inflammatory cytokines such as IL-4 and IL-13, with minimal disruption of pro-inflammatory markers in a pregnant mouse model (Hofbauer *et al.* 2025). In another study, T-cell–driven autoimmunity was effectively suppressed by a lipid NP–mediated delivery of an indoleamine-2,3-dioxygenase 1 (IDO1) mRNA variant (Kenney *et al.* 2024). In addition, as promising frameworks for targeted immune modulation, nanotherapeutic strategies that suppress IL-10 and IL-12 while increasing TGF-β release have been reviewed, which are aimed to promote tolerogenic immune responses (Scotland *et al.* 2023).

### Nanoparticle design

**Figure 1:**
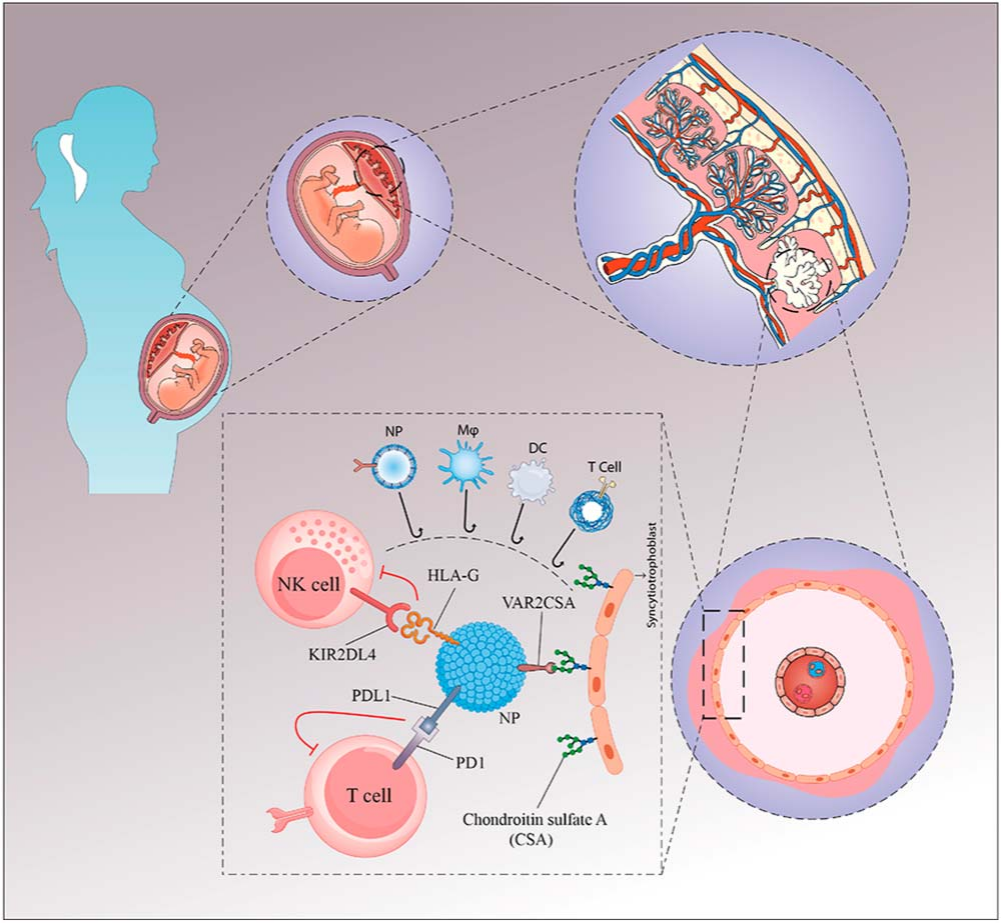
Schematic representation of nanoparticle-mediated immune cloaking at the maternal–fetal interface. By binding to chondroitin sulfate A (CSA), engineered nanoparticles (NPs) are functionalized with placental homing ligands, such as VAR2CSA, that target the syncytiotrophoblast layer. Once localized, NPs exhibit immune-modulatory molecules (e.g., PD-L1, HLA-G), which interact with immune cell receptors (PD-1 on T cells, KIR2DL4 on NK cells) to reduce cytotoxic activity and increase tolerance. Furthermore, NPs have the ability to alter dendritic cells (DCs) and macrophages (Mφ) to strengthen a tolerogenic milieu, which lowers the risk of fetal immune rejection.

According to Fig. 1, the basic components for biocompatibility and imaging are as follows:

- PLGA core: PLGA (poly lactic-co-glycolic acid) is a biodegradable polymer approved by the FDA that decomposes into lactic and glycolic acids (Makadia & Siegel 2011, Li *et al.* 2022). It has been employed in various drug formulations and medical devices, such as resorbable sutures, with its safety and degradation profiles well documented (Makadia & Siegel 2011). The PLGA core provides the NP with mechanical strength and can contain imaging materials or immunomodulating content if required.
- Lipid–PEG shell: the outer shell is composed of lipids that are conjugated to PEG. Lipid-PEG coatings are used to produce nanoparticles that are stealth in nature and avoid the MPS and opsonization of proteins (Torchilin 2004, Fam *et al.* 2020). PEG chains create a hydrophilic layer, which prolongs the circulation time of the NPs and improves the chances of delivering the NP to the placenta (Torchilin 2004). The lipid–polymer hybrid approach combines the robustness of polymeric nanoparticles with the deformability and compatibility of lipid vesicles (Torchilin 2004, Li *et al.* 2022).
- Superparamagnetic iron oxide nanoparticles (SPIONs): to enhance the non-invasive imaging capability, SPIONs can be blended into the PLGA core or adsorbed to the NP surface (Wáng & Idée 2017). SPIONs are T2-weighted contrast agents in MRI and help in the visualization of the NP distribution (Wáng & Idée 2017). This feature is very useful in the identification of the placenta and dose in preclinical studies; however, it can be omitted in clinical applications.

### Placental targeting ligands

Placental chondroitin sulfate A (plCSA) is expressed on the surface of syncytiotrophoblasts. A peptide from the VAR2CSA malaria protein that specifically binds these glycosaminoglycans has been identified (plCSA-binding peptide, plCSA-BP) (Zhang *et al.* 2018*a*). Previous studies have indicated that plCSA-BP–conjugated nanoparticles preferentially localize in the placenta with low off-target accumulation in maternal organs (Zhang *et al.* 2018*a*). This is important for preventing non-placental immune suppression and toxicity.

If VAR2CSA proves ineffective, alternative ligands can be targeted. For instance, small peptides including CGKRK and iRGD, previously identified for tumor probing, also exhibited high affinity toward placental vasculature (Hu *et al.* 2013, Zhang *et al.* 2018*b*). CGKRK-conjugated liposomes homed especially to spiral arteries and endovascular trophoblasts in mouse models, allowing for growth factor delivery that improved fetal outcomes (Hu *et al.* 2013). By including multiple peptides (plCSA-BP + CGKRK/iRGD), it is postulated that targeting specificity could be improved by targeting different placental sub-regions (Hu *et al.* 2013, Zhang *et al.* 2018*a*).

Regarding the relative targeting affinity/specificity and accumulation of these peptides, the plCSA-BP-conjugated NPs accumulate approximately six-fold more in the placenta than non-targeted controls according to prior research. They also show highly specific binding to trophoblast cells and minimal systemic or fetal exposure (Tang *et al.* 2023). On the other hand, tumor-homing peptides CGKRK and iRGD are approximately eight- and seven-fold more abundant in placental tissue than in other maternal organs, respectively (King *et al.* 2016). They bind particularly to the syncytiotrophoblast layer, spiral arteries, and the placental labyrinth in both mice and human explants (King *et al.* 2016, Tang *et al.* 2023). Therefore, combining plCSA-BP and CGKRK/iRGD may improve complementary binding mechanisms, since plCSA-BP provides trophoblast specificity and CGKRK/iRGD targets vascular regions, which can improve overall placental targeting efficacy.

### Immune cloaking

Immune cloaking refers to a biological tactic whereby cells, tissues, or therapeutics mask or alter the presentation of antigens to evade recognition by the host immune system (Mohale *et al.* 2022). Immunomodulatory factors, immune checkpoint ligand expression, and downregulation of major histocompatibility complex molecules are all examples of immune cloaking during pregnancy, which prevent the mother’s immune system from rejecting the semi-allogeneic fetus (Yousefzadeh *et al.* 2022). These mechanisms have similarities in the biology of transplantation and cancer immune evasion and are essential for preserving immune tolerance at the maternal–fetal interface.

Previous studies have shown that NPs can be cleared from the body by macrophages and the reticuloendothelial system (liver and spleen), reducing circulation time and delivery to target tissues (Gustafson *et al.* 2015, Li *et al.* 2019). To overcome this challenge, we can use immune cloaking with core strategies that include: PEG per se inhibits NP uptake by macrophages and reduces unwanted immune activation (Torchilin 2004, Fam *et al.* 2020). In addition, coating NPs with red blood cell (RBC) membranes adds a ‘self’ marker (CD47) that inhibits macrophage phagocytosis, which prolongs circulation time and reduces clearance (Li *et al.* 2019, Xia *et al.* 2019). Furthermore, future versions may link HLA-G or PD-L1 fragments to bind inhibitory receptors on NK and T cells. Nevertheless, this approach is complicated and expensive.

Together, these mechanisms might allow the placenta to escape an immune attack from an over-reactive maternal immune system (Fig. 1). However, this strategy does have certain drawbacks. Isolating and coating membranes on a large scale are still technically challenging and expensive. Furthermore, RBC membrane-coated NPs have the potential to be immunogenic, especially if they originate from allogeneic or mismatched donors. Autologous or universally compatible donor RBCs, standardized membrane purification processes, and scalable microfluidic coating technologies can aid in overcoming these obstacles (Li *et al.* 2023). Consequently, despite practical challenges, RBC membrane cloaking remains a promising strategy for enhancing NP circulation and reducing immune clearance.

**Figure 2:**
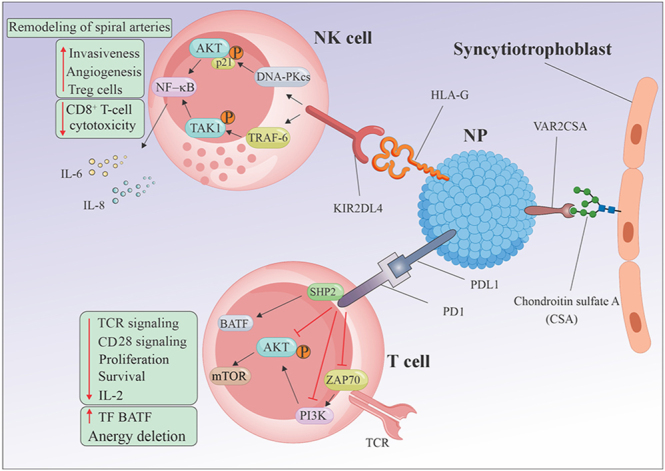
The proposed mechanism describes how HLA-G interacts with NK cells to facilitate spiral artery remodeling and the functions of the PD-1/PD-L1 pathway on T cells. HLA-G binds to the KIR2DL4 receptor on NK cells. The KIR2DL4 receptor associates with TRAF6, leading to TAK1 phosphorylation and activation of the NF-κB pathway. Concurrently, KIR2DL4 engages with DNA-PKcs, initiating Akt phosphorylation and subsequently increasing p21 expression. The phosphorylated Akt further stimulates the NF-κB pathway, inducing the senescence-associated secretory phenotype (SASP). As a result, SASP-related factors such as IL-6 and IL-8 are produced, which enhance vascular permeability, support angiogenesis, and promote tissue invasion. PD-1 suppresses T cell activity by recruiting phosphatases, particularly SHP2. These phosphatases antagonize the stimulatory signals generated through T cell receptor (TCR) engagement with MHCI and CD28 interaction with CD80 and/or CD86. Specifically, they inhibit key signaling components, such as ZAP70, as well as the phosphoinositide 3-kinase (PI3K)–AKT pathway. The cumulative effect is a reduction in the activation of transcription factors, including mTOR and BATF, which are critical for T cell activation, proliferation, effector function, and survival.

Engagement of the T cell receptor (TCR) together with CD3/CD28 co-stimulation initiates intracellular signaling cascades characterized by activation of phosphatidylinositol 3-kinase (PI3K), which subsequently triggers protein kinase B (AKT) activation (Fig. 2). The PI3K/AKT pathway promotes T cell proliferation and cytokine production (Zhang *et al.* 2015). In contrast, PD-1 interferes with membrane-proximal signaling in T cells by suppressing PI3K activation and the subsequent activation of AKT. It reduces the phosphorylation of zeta-chain-associated protein 70 kDa (ZAP-70) and protein kinase θ (PKCθ), and suppresses the activation of extracellular signal-regulated kinase (ERK). Ultimately, PD-1 engagement promotes T-cell exhaustion by diminishing cytokine production and impairing T-cell proliferation and survival (Sharpe & Pauken 2018).

NK cells express the KIR2DL4 receptor, which specifically recognizes HLA-G (Fig. 2). The interaction between HLA-G and KIR2DL4 is pivotal for establishing immune tolerance at the maternal–fetal interface (Djurisic *et al.* 2015). HLA-G exhibits similar functions by suppressing the cytotoxic activity of decidual NK (dNK) cells and modulating spiral artery remodeling as well as fetal growth. HLA-G can suppress NK cell and CD8^+^ T-cell cytotoxicity while increasing the proportion of Treg cells, thereby promoting immune tolerance (Xu *et al.* 2020, Prašnikar *et al.* 2022). The signal produced through the interaction of HLA-G with KIR2DL4 receptors on maternal–fetal NK cells leads to the secretion of key factors, including IL-6 and IL-8, which promote and reinforce the senescent state. These senescence-associated signals contribute to vascular remodeling by enhancing endothelial permeability (Rajagopalan *et al.* 2014).

### Scalable production methods

**Figure 3:**
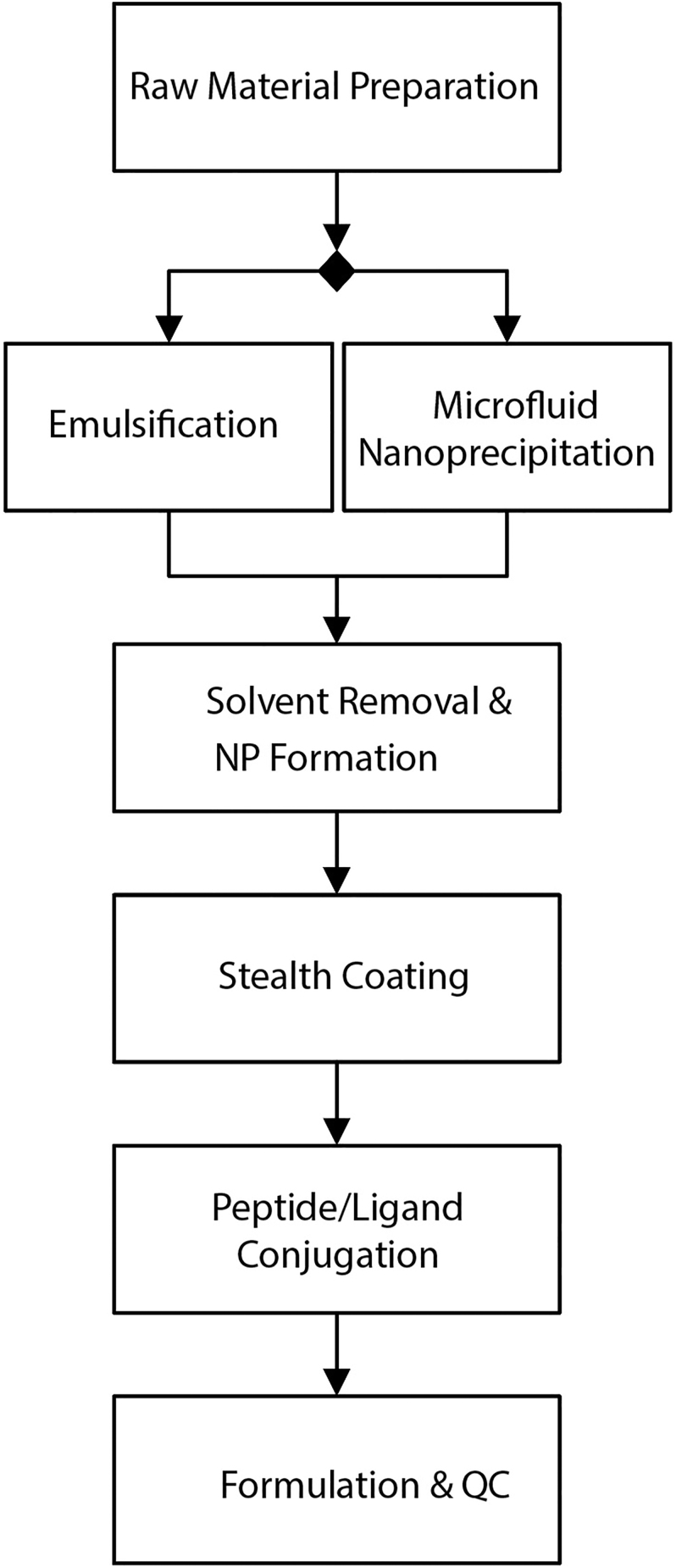
Flowchart summarizing the large-scale manufacturing steps (emulsion–solvent evaporation or microfluidic nanoprecipitation).

Well-established methods of emulsion–solvent evaporation and nanoprecipitation can yield PLGA-based NPs at scale (Tetyczka *et al.* 2022). In a typical procedure, PLGA and SPIONs are dissolved in a volatile organic solvent (e.g. dichloromethane) to form the oil phase. This is emulsified in an aqueous phase containing a stabilizer (such as polyvinyl alcohol). As the droplet size becomes smaller and the internal pressure of the droplet increases, the solvent evaporates. Because the solvent stops any further mixing, solid NPs are formed. In another method, the same procedure is performed using a microfluidic device. The microfluidic procedure achieves narrower size distributions. It is also highly amenable to industrial upscaling (Tetyczka *et al.* 2022). Figure 3 demonstrates the flowchart of large-scale manufacturing steps.

## Proposed experimental validation

### *In vitro* assays

- Trophoblast binding: fluorescently labeled and peptide-functionalized NPs will be incubated with human trophoblast cell lines (e.g. HTR-8/SVneo) and cell lines of decidual cells to assess binding specificity. We expect higher uptake in trophoblasts compared to non-targeted controls (Zhang *et al.* 2018*a*).
- Immune evasion: uptake of RBC membrane–coated and uncoated NPs will be quantified in phagocytosis assays with macrophage-like cells (e.g. THP-1). Reduced uptake of the membrane–coated NPs will indicate that these NPs are effectively cloaked from the immune system.
- Cytotoxicity and immunogenicity: MTT or AlamarBlue assays will determine NP cytotoxicity in trophoblasts, endothelial cells, and maternal immune cells. Complement activation assays in human plasma will serve to confirm minimal off-target immune activation (Li *et al.* 2022).
- Co-culture with immune cells: to assess how NPs (alone or trophoblast-pretreated) affect maternal immune responses, trophoblast–immune cell co-cultures will be established using HTR-8/SVneo or primary cytotrophoblasts with monocyte-derived macrophages, decidual/peripheral NK cells, and T cells. Readouts will include immune cell viability, activation/polarization markers, NK degranulation, T-cell proliferation, cytokine/chemokine secretion, complement activation, and NP localization. Macrophage phagocytosis will be quantified via flow cytometry and live imaging of fluorescent NPs.

Overall, the *in vitro* study will help us edit and optimize the NP structure to achieve the best possible outcome.

### *In vivo* studies

- Biodistribution and MRI tracking: SPION-loaded, fluorescent NPs will be injected intravenously into pregnant mice. *In vivo* optical imaging (IVIS) and *ex vivo* organ fluorescence quantification will identify the localization of the NPs. T2 MRI with SPION enhancement will confirm placental accumulation and retention (Wáng & Idée 2017, Zhang *et al.* 2018*a*).
- Efficacy in immune-mediated RPL models:
  ○ CBA/J × DBA/2 mating model: this model has a high embryo resorption rate because of Th1-biased immune responses. Testing it with NPs will help elucidate whether early gestational treatments can target the immune response and reduce this resorption.
  ○ Anti-paternal sensitization model: females who are pre-sensitized to paternal antigens show increased miscarriage frequency. The timing and dosage of the NP injection will be optimized to see if placental tolerance is restored in pre-sensitized females.
- Safety and developmental assessments: maternal health, fetal weight and growth, and histopathology of major organs (liver, spleen, and placenta) will be monitored. Ultrasound imaging of the fetus will detect any potential teratogenic effects. Previous studies have indicated minimal fetal exposure to placental-targeted NPs (Zhang *et al.* 2018*a*,*b*, Tetyczka *et al.* 2022).

### Clinical trial design

Initial human testing will begin with a phase I clinical trial focused primarily on safety and tolerability in a small cohort of women with a history of uRPL who have no viable treatment alternatives (Practice Committee of the American Society for Reproductive Medicine 2012). We will use our experience in previous immunotherapy clinical trials of RPL patients (Aslanian-Kalkhoran *et al.* 2023, Dashti *et al.* 2025) to select patients with immune-related miscarriage with the following criteria and dosing regimen:

#### Inclusion criteria

- Women aged 18–40 years.
- Women who experienced two or more consecutive pregnancy losses before reaching 20 weeks of gestation, with or without previous pregnancies of 20 weeks or longer.
- No evidence of major fetal structural or chromosomal abnormalities.
- Willingness to provide informed consent and comply with study visits.

#### Exclusion criteria

- Participants with a history of prior immunological treatment, infertility, uterine anomalies, endocrine disorder (e.g. thyroid dysfunction, diabetes mellitus), polycystic ovarian syndrome, chronic endometritis, acquired and inherited thrombophilia, antithrombin deficiency, chromosomal abnormality, autoimmune or immunodeficiency conditions, and abnormalities of autoantibodies.
- Known hypersensitivity to PLGA, PEG, iron oxide, or ligand peptides.

#### Dosing regimen

Phase I will use a 3 + 3 dose-escalation format to determine the maximum tolerated dose (MTD), with 100% increments of escalation starting at 0.05 mg/kg NP-equivalent. For a maximum of four doses, intravenous infusions will be given every 2 weeks. Phase II will make use of the MTD or biologically active dose.

#### Key endpoints will include

- Maternal safety: during and after infusion, mothers will be monitored for serious adverse events, including infusion reactions, complement activation, and hepatic/renal function.
- Fetal safety: initial ultrasound and Doppler assessments will identify any abnormal fetal growth.
- Placental localization: optional MRI or specialized ultrasound with contrast will confirm NP localization.

Phase II trials will assess efficacy in a larger sample size (∼30 participants per arm) after safety and dose parameters have been established. These trials will focus on clinically relevant outcomes, including the rate of live births, the decrease in the incidence of miscarriages, and improvements in immune biomarkers such as immune cell activity (NK cells and T helpers) and cytokine profiles (Practice Committee of the American Society for Reproductive Medicine 2012, Aslanian-Kalkhoran *et al.* 2023, Dashti *et al.* 2025). A randomized, controlled design will be used in this phase to compare NP therapy to either a placebo or standard care.

A successful phase II result would support a phase III pivotal trial intended to verify safety and efficacy across several centers, with enough power to identify significant variations in long-term maternal and fetal health outcomes, as well as live birth rates. Good manufacturing practices (GMP) validation, the submission of an investigational new drug application, and a thorough safety data review are all examples of regulatory engagement with organizations such as the Food and Drug Administration (FDA) or Therapeutic Goods Administration (TGA). The process of gaining clinical approval will be sped up by early regulatory body consultation, which will help align trial design and endpoints.

Manufacturing procedures will adhere to GMP during development to guarantee the NP formulations’ consistent quality and safety. After approval, post-market surveillance and long-term safety monitoring will be scheduled to evaluate uncommon adverse events and practical efficacy.

#### Statistical considerations

- Phase I: descriptive statistics for safety outcomes; MTD determined by dose-limiting toxicities in ≤1 of 6 participants per cohort.
- Phase II: sample size calculated to detect a minimum 20% improvement in fetal growth velocity (*α* = 0.05, power = 80%); assuming SD = 25%, ∼30 participants per arm will be required.
- Between-group comparisons will be performed using ANCOVA, adjusting for baseline growth velocity.
- Missing data will be handled with multiple imputation where appropriate.

According to preclinical data, targeted nanoparticle therapy significantly decreased miscarriage rates by up to 40% in murine models (Wang *et al.* 2024). If these effects translate to humans, even a conservative 20% reduction in RPL incidence could result in substantial improvements in live birth rates, which would benefit thousands of impacted couples each year. Such a reduction would be a significant clinical benefit, outperforming the inconsistency of current systemic immunosuppressive therapies (Parhizkar *et al.* 2022, Yang *et al.* 2022, Aslanian-Kalkhoran *et al.* 2023, The ESHRE Guideline Group on RPL *et al.* 2023, Dashti *et al.* 2025). Validation of these projections will require additional clinical trials.

### Cost-effectiveness

Since all the basic materials – PLGA, lipids, PEG, and iron oxide – have already been used in medical products, the cost per dose stays low. PLGA and iron oxide are bulk commodities. Lipid-PEG mixtures and targeting peptides constitute a higher fraction of the overall cost per dose; these are administered in microgram to milligram quantities.

Even with some markup for formulation and quality control, the projected final cost to produce an injectable product remains substantially lower than that of recombinant protein-based therapies or repeated IVIG infusions (Table 1).

**Table 1:** Estimated material costs per dose.

Component	Approx. Cost range per dose	Comment
PLGA	$0.01–$0.05	Bulk production; FDA-approved
Iron oxide (SPION)	$0.01–$0.03	Industrial-grade magnetite is low-cost
Lipid–PEG	$0.30–$3.00	Costly per gram, but used in very small quantities
Targeting peptides	$0.20–$2.00	Synthetic peptide costs vary; used sparingly
RBC membrane/stealth	$0.05–$0.50	If donor RBC membrane is used; minimal cost
Total	$0.56–$5.58	Significantly cheaper than IVIG or biologics

PLGA, poly lactic-co-glycolic acid; PEG, polyethylene glycol; SPION, superparamagnetic iron oxide nanoparticles; RBC, red blood cell; FDA, Food and Drug Administration; IVIG, intravenous immunoglobulin.

## Safety considerations and translational risk mitigation

Although the NP platform uses validated materials, safety will be systematically addressed:

- Off-target effects: liver and spleen uptake will be quantified; ligand density and NP size optimized to minimize reticuloendothelial clearance (Gupta & Gupta 2005, Wáng & Idée 2017).
- Long-term fate: PLGA biodegradation products are naturally metabolized (Makadia & Siegel 2011, Gentile *et al.* 2014). SPION iron load will be monitored via MRI and biochemical assays (Soenen *et al.* 2011).
- Immunogenicity: anti-PEG antibodies and potential peptide immunogenicity will be evaluated *in vitro* and in repeated-dose animal studies (Verhoef & Anchordoquy 2013, Kozma *et al.* 2020).
- Monitoring plan:
  ○ In preclinical models: maternal/fetal histology, biodistribution, clearance kinetics, and antibody titers.
  ○ In clinical trials: slow infusion protocols, real-time monitoring, and serial immune biomarker panels.

## Limitations

One of the primary limitations of our design lies in the functional outcomes of the selected components. For instance, the use of specific placental ligands such as VAR2CSA and the incorporation of magnetic elements such as SPIONs may present challenges, including potential adverse effects or limited clinical applicability. However, the modular nature of the nanoparticle design allows for flexibility; these components can be modified, replaced, or removed entirely to optimize performance and safety for clinical translation.

Moreover, large-scale nanoparticle production poses challenges, particularly in achieving consistent quality from batch to batch, with each batch required to match previous and subsequent batches as closely as possible. We need each batch to be nearly identical to the previous batch and to the next one. We judge quality by measuring three key attributes: i) size, ii) surface charge, and iii) peptide density. If, for some reason, we were to allow more variation in size, then the next key attribute should be much more consistent and should be much more easily attainable – and that’s the surface charge.

## Conclusion and future directions

Current management of RPL relies on systemic immunosuppression, offering inconsistent efficacy and exposing mothers and fetuses to substantial risk. Our proposed placenta-targeted nanoparticle platform represents a paradigm shift – enabling precise immune modulation at the maternal–fetal interface without compromising systemic immunity. By integrating FDA-approved materials with placenta-specific ligands and immune-cloaking surfaces, this strategy delivers a clinically translatable, cost-effective, and scalable solution.

If validated, this approach has the potential to not only reduce miscarriage rates but also transform the management of other placenta-mediated disorders, including preeclampsia and fetal growth restriction. Achieving this vision will require coordinated translational research, strategic investment, and multidisciplinary collaboration. The development of safe, targeted nanomedicine for pregnancy could redefine therapeutic possibilities in reproductive medicine and bring tangible hope to families worldwide.

## Declaration of interest

The authors declare that a provisional patent application (Application No. 2025900687, filing date: 07 Mar 2025) has been filed covering the design, composition, and therapeutic use of the placenta-targeted nanoparticle platform described in this work.

## Funding

This research did not receive any specific grant from any funding agency in the public, commercial, or not-for-profit sector.

## Author contribution statement

MD was responsible for visualization, writing the original draft, and writing review and editing. AA was responsible for conceptualization, investigation, visualization, and writing the original draft. MY contributed to conceptualization, methodology, writing the original draft, supervision, project administration, and writing review and editing. All authors have contributed to the research and approved its final version for submission.
